# Characterization of Posa and Posa-like virus genomes in fecal samples from humans, pigs, rats, and bats collected from a single location in Vietnam

**DOI:** 10.1093/ve/vex022

**Published:** 2017-08-23

**Authors:** Bas B. Oude Munnink, My V.T. Phan, The VIZIONS Consortium, Peter Simmonds, Marion P.G. Koopmans, Paul Kellam, Lia van der Hoek, Matthew Cotten

**Affiliations:** 1 Department of Virus Genomics, Wellcome Trust Sanger Institute, Hinxton CB10 1SA, UK; 2 Department of Viroscience, Erasmus Medical Center, Rotterdam, The Netherlands; 3 Nuffield Department of Medicine, University of Oxford, Oxford OX1 3SY, UK; 4 Department of Infectious Diseases and Immunity, Imperial College London, London, UK; 5 Laboratory of Experimental Virology, Academic Medical Center, University of Amsterdam, Amsterdam, The Netherlands

**Keywords:** virus discovery, next generation sequencing, husavirus, posavirus, rasavirus, basavirus, *Picornavirales*

## Abstract

Porcine stool-associated RNA virus (posavirus), and Human stool-associated RNA virus (husavirus) are viruses in the order *Picornavirales* recently described in porcine and human fecal samples. The tentative group (Posa and Posa-like viruses: PPLVs) also includes fish stool-associated RNA virus (fisavirus) as well as members detected in insects (*Drosophila subobscura* and *Anopheles sinensis*) and parasites (*Ascaris suum*). As part of an agnostic deep sequencing survey of animal and human viruses in Vietnam, we detected three husaviruses in human fecal samples, two of which share 97–98% amino acid identity to Dutch husavirus strains and one highly divergent husavirus with only 25% amino acid identity to known husaviruses. In addition, the current study found forty-seven complete posavirus genomes from pigs, ten novel rat stool-associated RNA virus genomes (tentatively named rasavirus), and sixteen novel bat stool-associated RNA virus genomes (tentatively named basavirus). The five expected *Picornavirales* protein domains (helicase, 3C-protease, RNA-dependent RNA polymerase, and two Picornavirus capsid domain) were found to be encoded by all PPLV genomes. In addition, a nucleotide composition analysis revealed that the PPLVs shared compositional properties with arthropod viruses and predicted non-mammalian hosts for all PPLV lineages. The study adds seventy-six genomes to the twenty-nine PPLV genomes currently available and greatly extends our sequence knowledge of this group of viruses within the *Picornavirales* order.

## 1. Introduction

The order *Picornavirales* includes a wide range of viruses that infect a variety of hosts. According to the latest International Committee on Taxonomy of Viruses (ICTV) classification (ICTV 2017), the order comprises five families: the families *Dicistroviridae* and *Iflaviridae* contain members which infect insects (e.g. cripavirus and deformed wing virus), the family *Secoviridae* members which infect plants (e.g. turnip ringspot virus), the family *Picornaviridae* members infecting vertebrates (e.g. enteroviruses) and the family *Marnaviridae.* The latter contains only *Heterosigma akashiwo* virus for which algae is the natural host (Le Gall et al. 2008).

Although members of the *Picornavirales* are highly diverse, they share a number of common features, including a single stranded positive-sense RNA genome and co-linear genes encoding a helicase, protease, and RNA-dependent RNA polymerase (RdRP) replication block (Le Gall et al. 2008). The genome lengths for *Picornavirales* range from 7.2 to 9.8 kb. Typically, the encoded polyprotein is cleaved by virus-encoded proteases (Blom et al. 1996). Generally, members of the *Picornavirales* are monopartite, although some members of the *Secoviridae* have genomes with two segments (Le Gall et al. 2008).

Increasing improvements in next-generation sequencing (NGS) has identified a number of divergent members of the order *Picornavirales*. Porcine stool-associated RNA viruses (posaviruses) were found in the feces of healthy pigs and water collected from swine farms (Shan et al. 2011, Hause et al. 2015, 2016), fish stool-associated RNA virus (fisavirus) was identified in the intestinal content of a healthy carp (Reuter et al. 2015), and human stool-associated RNA virus (husavirus) was identified in the feces of predominantly healthy humans (Oude Munnink et al. 2015). Although structually closely related (based on the genome organisation), these viruses display broad genetic diversity with often less than 40% amino acid identity in specific coding regions thereby suggesting a deep evolutionary history of the virus family.

Although posaviruses can be detected at high frequency in pig fecal samples (21%), a recent study using immunoprecipitation coupled with PCR detection assay showed that posavirus antibodies were infrequently detected (Hause et al. 2016). The possibility that posaviruses may not infect pigs but rather infect gut commensal organisms or have a dietary or environmental origin is supported by blast analysis of posavirus sequences that showed that some posavirus strains have greatest sequence similarity to an RNA sequence from the parasite *Ascaris suum* (Shan et al. 2011; Wang et al. 2011). Furthermore, a mRNA sequence from the mosquito *Anopheles sinensis* and a virus recently identified in the fruit fly *Drosophila subobscura* have been described showing some sequence identity to posaviruses (Webster et al. 2016). Although the viruses have been identifed in samples from different hosts, the true infection hosts for fisavirus, posavirus, and husavirus remain to be determined.

As part of a study to define patterns of viral zoonosis in Vietnam (Rabaa et al. 2015), we performed detailed agnostic (random-primed) whole-genome deep sequencing (Cotten et al. 2014) on fecal samples from bats, humans, pigs, and rats and rectal swabs from humans and pigs. We have analyzed these sequence data for the presence of PPLVs and we describe here a large set of novel virus genomes from human, rat, pig, and bat samples that share homology and protein domain architecture with the previous described posaviruses.

## 2. Results

For simplicity, we will use the term Posa and Posa-like viruses (PPLVs) throughout the manuscript. The PPLV category comprises virus and virus sequences that show >30% amino acid homology to the existing posavirus and husavirus genome sequences, do not cluster within the five established *Picornavirales* families and show a *Picornavirales* genome organization with the expected five *Picornavirales* protein domains (see below for further details). A search for PPLV genomes in sequences was performed as follows: short read data (3–4 million 250 nt paired end reads per sample) were *de novo* assembled into longer sequence contigs and a protein sequence based USEARCH analysis (Edgar 2010) was performed against a database containing all *Picornavirales* protein entries in GenBank, including all known posaviruses sequences. This search identified three husaviruses, forty-seven posaviruses, ten novel rasaviruses, and sixteen novel basaviruses genomes. The genome lengths of the newly identified PPLV genome sequences varied from 8,262 to 11,318 nucleotides and for all viruses the read coverage across the genome and G + C content was determined. The results of these analyses and the available demographical data for these samples are summarized in Table 1.

**Table 1.** Overview of the PPLVs identified in this study.

Illumina ID	Accession number	ENA number lane 1	ENA number lane 2	Lineage	Sample source	Sample type	Source age	Date of sample	Genome length	G + C content (%)	Median depth of coverage^a^
Basavirus_16715_47	KX673228	ERR1301485	ERR1301574	Bv_7	*Scotophilus kuhlii*	Fecal	Unknown	10 Jun 2014	9,591	36.7	116
Basavirus_16715_47_2	KX673229	ERR1301485	ERR1301574	Bv_3	*Scotophilus kuhlii*	Fecal	Unknown	17 Jun 2014	9,585	32.1	42
Basavirus_16715_5	KX673230	ERR1301446	ERR1301535	Bv_6	*Scotophilus kuhlii*	Fecal	Unknown	12 Nov 2014	8,776	40.3	13
Basavirus_16715_61	KX673234	ERR1301499	ERR1301588	Bv_7	*Scotophilus kuhlii*	Fecal	Unknown	11 Jun 2014	9,566	36.6	129
Basavirus_16715_61_2	KX673235	ERR1301499	ERR1301588	Bv_6	*Scotophilus kuhlii*	Fecal	Unknown	11 Jun 2014	9,065	40.3	208
Basavirus_16715_69	KX673237	ERR1301507	ERR1301596	Bv_3	*Scotophilus kuhlii*	Fecal	Unknown	11 Jun 2014	9,528	32.2	21
Basavirus_16715_71	KX673238	ERR1301509	ERR1301598	Bv_7	*Scotophilus kuhlii*	Fecal	Unknown	11 Jun 2014	9,585	36.9	132
Basavirus_16715_71_2	KX673239	ERR1301509	ERR1301598	Bv_3	*Scotophilus kuhlii*	Fecal	Unknown	11 Jun 2014	9,483	32.1	29
Basavirus_16715_77	KX673240	ERR1301514	ERR1301603	Bv_1	*Scotophilus kuhlii*	Fecal	Unknown	10 Jun 2014	8,829	38.6	28
Basavirus_16715_84	KX673241	ERR1301519	ERR1301608	Bv_7	*Scotophilus kuhlii*	Fecal	Unknown	17 Jun 2014	9,391	36.9	48
Basavirus_16715_86	KX673242	ERR1301521	ERR1301610	Bv_3	*Scotophilus kuhlii*	Fecal	Unknown	17 Jun 2014	9,530	32.2	94
Basavirus_16845_64	KX673243	ERR1301829	ERR1301914	Bv_3	*Scotophilus kuhlii*	Fecal	Unknown	18 Sep 2014	9,537	32.2	160
Basavirus_16845_79	KX673244	ERR1301842	ERR1301927	Bv_2	*Scotophilus kuhlii*	Fecal	Unknown	18 Sep 2014	9,147	35.9	55
Basavirus_16845_94	KX673245	ERR1301855	ERR1301940	Bv_4	*Scotophilus kuhlii*	Fecal	Unknown	17 Sep 2014	8,262	48.2	27
Basavirus_17819_2	KX673289	ERR1302928	ERR1303004	Bv_5	*Scotophilus kuhlii*	Fecal	Unknown	12 Nov 2014	9,271	46.9	171
Basavirus_17819_8	KX673290	ERR1302934	ERR1303010	Bv_5	*Scotophilus kuhlii*	Fecal	Unknown	12 Nov 2014	9,240	47.1	274
Husavirus_16370_59	KX673221	ERR1301365	ERR1301415	Hv_1	*Homo sapiens*	Fecal	7 years	2 Apr 2013	8,856	52.8	10
Husavirus_16915_89	KX673248	ERR1302009	ERR1302077	Hv_2	*Homo sapiens*	Rectal swab	51 years	24 Mar 2013	8,576	50.8	17
Husavirus_19344_29	KX673274	ERS1725523	ERS1725523	Hv_1	*Homo sapiens*	Fecal	59 years	2 Oct 2014	9,027	53	97
Posavirus_12087_40	KX673215	ERR473400	NA	Pv_6	*Sus domesticus*	Fecal	1 month	12 Mar 2012	11,036	43.8	32
Posavirus_12087_42	KX673216	ERR473402	NA	Pv_6	*Sus domesticus*	Fecal	1.5 months	14 Mar 2012	11,318	44.1	24
Posavirus_12144_61	KX673217	ERR477293	NA	Pv_1	*Sus domesticus*	Fecal	20 months	10 Apr 2012	9,729	35.8	135
Posavirus_14226_38	KX673218	ERR775480	NA	Pv_3	*Sus domesticus*	Fecal	15 months	6 Mar 2012	9,190	46.5	34
Posavirus_14226_39	KX673219	ERR775481	NA	Pv_3	*Sus domesticus*	Fecal	15 months	6 Mar 2012	9,071	46.4	25
Posavirus_14250_11	KX673220	ERR779984	NA	Pv_5	*Sus domesticus*	Fecal	2 months	21 Mar 2012	9,680	33.2	55
Posavirus_16915_3	KX673246	ERR1301944	ERR1302014	Pv_3	*Sus domesticus*	Rectal swab	5 months	19 Mar 2013	9,296	46.8	45
Posavirus_16915_5	KX673247	ERR1301946	ERR1302016	Pv_3	*Sus domesticus*	Rectal swab	5 months	19 Mar 2013	9,204	46.7	51
Posavirus_17489_2	KX673249	ERR1302404	ERR1302484	Pv_1	*Sus domesticus*	Rectal swab	5 months	27 Mar 2013	9,835	36.5	127
Posavirus_17489_26	KX673250	ERR1302422	ERR1302502	Pv_1	*Sus domesticus*	Rectal swab	5 months	3 Apr 2013	9,827	36.4	111
Posavirus_17489_27	KX673251	ERR1302423	ERR1302503	Pv_1	*Sus domesticus*	Rectal swab	5 months	3 Apr 2013	9,773	36.5	16
Posavirus_17489_28	KX673252	ERR1302424	ERR1302504	Pv_1	*Sus domesticus*	Rectal swab	5 months	3 Apr 2013	9,824	36.4	65
Posavirus_17489_30	KX673253	ERR1302426	ERR1302506	Pv_1	*Sus domesticus*	Rectal swab	5 months	3 Apr 2013	9,672	36.4	193
Posavirus_17489_34	KX673254	ERR1302428	ERR1302508	Pv_5	*Sus domesticus*	Rectal swab	5 months	3 Apr 2013	8,970	31.5	2184
Posavirus_17489_35	KX673255	ERR1302429	ERR1302509	Pv_5	*Sus domesticus*	Rectal swab	5 months	3 Apr 2013	9,733	33.5	146
Posavirus_17489_36	KX673256	ERR1302430	ERR1302510	Pv_5	*Sus domesticus*	Rectal swab	5 months	3 Apr 2013	9,729	33.5	842
Posavirus_17489_39	KX673257	ERS1725815	ERS1725815	Pv_5	*Sus domesticus*	Rectal swab	5 months	3 Apr 2013	9,640	33.5	20
Posavirus_17489_4	KX673258	ERR1302405	ERR1302485	Pv_4	*Sus domesticus*	Rectal swab	5 months	27 Mar 2013	8,970	31.5	29
Posavirus_17489_4_2	KX673259	ERR1302405	ERR1302485	Pv_1	*Sus domesticus*	Rectal swab	5 months	27 Mar 2013	9,647	36.4	5
Posavirus_17489_40	KX673260	ERR1302433	ERR1302513	Pv_5	*Sus domesticus*	Rectal swab	5 months	3 Apr 2013	9,650	33.5	70
Posavirus_17489_45	KX673261	ERR1302438	ERR1302518	Pv_5	*Sus domesticus*	Rectal swab	5 months	4 Apr 2013	9,710	33.2	18
Posavirus_17489_47	KX673262	ERR1301975	ERR1302045	Pv_5	*Sus domesticus*	Rectal swab	5 months	4 Apr 2013	9,783	33.2	24
Posavirus_17489_5	KX673263	ERR1302406	ERR1302486	Pv_4	*Sus domesticus*	Rectal swab	5 months	27 Mar 2013	9,222	31.2	101
Posavirus_17489_50	KX673264	ERR1302440	ERR1302520	Pv_6	*Sus domesticus*	Rectal swab	5 months	4 Apr 2013	11,094	44.6	45
Posavirus_17489_51	KX673265	ERR1302441	ERR1302521	Pv_4	*Sus domesticus*	Rectal swab	2 years	4 Apr 2013	9,286	31.4	309
Posavirus_17489_60	KX673266	ERR1302450	ERR1302530	Pv_5	*Sus domesticus*	Rectal swab	5 months	4 Apr 2013	9,859	33.4	39
Posavirus_17489_7	KX673267	ERR1302407	ERR1302487	Pv_1	*Sus domesticus*	Rectal swab	5 months	27 Mar 2013	9,434	36.3	12
Posavirus_17489_86	KX673268	ERR1302473	ERR1302553	Pv_3	*Sus domesticus*	Rectal swab	5 months	18 Apr 2013	9,283	46.7	237
Posavirus_17489_87	KX673269	ERR1302474	ERR1302554	Pv_3	*Sus domesticus*	Rectal swab	5 months	18 Apr 2013	9,242	46.7	14
Posavirus_17489_90	KX673270	ERR1302477	ERR1302557	Pv_3	*Sus domesticus*	Rectal swab	5 months	18 Apr 2013	8,469	46.6	14
Posavirus_17489_91	KX673271	ERR1302478	ERR1302558	Pv_3	*Sus domesticus*	Rectal swab	5 months	18 Apr 2013	9,272	46.9	66
Posavirus_17489_95	KX673272	ERR1302481	ERR1302561	Pv_4	*Sus domesticus*	Rectal swab	5 months	18 Apr 2013	9,216	30	153
Posavirus_17489_95_2	KX673273	ERR1302481	ERR1302561	Pv_5	*Sus domesticus*	Rectal swab	5 months	18 Apr 2013	9,570	32.8	47
Posavirus_17668_10	KX673276	ERR1302758	ERR1302847	Pv_3	*Sus domesticus*	Rectal swab	5 months	4 May 2013	9,309	46.6	154
Posavirus_17668_11	KX673277	ERR1302759	ERR1302848	Pv_1	*Sus domesticus*	Rectal swab	5 months	4 May 2013	9,855	36.5	200
Posavirus_17668_11_2	KX673278	ERR1302759	ERR1302848	Pv_4	*Sus domesticus*	Rectal swab	5 months	4 May 2013	9,066	31.2	18
Posavirus_17668_12	KX673279	ERR1302760	ERR1302849	Pv_3	*Sus domesticus*	Rectal swab	5 months	4 May 2013	9,196	46	38
Posavirus_17668_13	KX673280	ERR1302761	ERR1302850	Pv_5	*Sus domesticus*	Rectal swab	5 months	4 May 2013	9,666	33.4	31
Posavirus_17668_13_2	KX673281	ERR1302761	ERR1302850	Pv_3	*Sus domesticus*	Rectal swab	5 months	4 May 2013	9,111	46.1	52
Posavirus_17668_33	KX673282	ERR1302780	ERR1302869	Pv_6	*Sus domesticus*	Rectal swab	6 months	16 Oct 2013	11,171	43.5	78
Posavirus_17668_33_2	KX673283	ERR1302780	ERR1302869	Pv_3	*Sus domesticus*	Rectal swab	6 months	16 Oct 2013	9,289	46.3	26
Posavirus_17668_33_3	KX673284	ERR1302780	ERR1302869	Pv_9	*Sus domesticus*	Rectal swab	6 months	16 Oct 2013	9,498	47.1	77
Posavirus_17668_4	KX673275	ERR1302752	ERR1302841	Pv_5	*Sus domesticus*	Rectal swab	5 months	4 May 2013	9,651	33.5	30
Posavirus_17668_47	KX673285	ERR1302794	ERR1302883	Pv_6	*Sus domesticus*	Rectal swab	5 months	16 Oct 2013	11,295	45.1	99
Posavirus_17668_71	KX673286	ERR1302816	ERR1302905	Pv_7	*Sus domesticus*	Rectal swab	6 months	17 Oct 2013	9,404	46.3	47
Posavirus_17668_83	KX673287	ERR1302827	ERR1302916	Pv_7	*Sus domesticus*	Rectal swab	5 months	17 Oct 2013	9,366	46.9	46
Posavirus_17668_86	KX673288	ERR1302830	ERR1302919	Pv_7	*Sus domesticus*	Rectal swab	5 months	17 Oct 2013	9,377	46.8	42
Rasavirus_16715_12	KX673222	ERR1301453	ERR1301542	Rv_2	*Rattus argentiventer*	Fecal	Unknown	12 Nov 2014	9,601	40.2	251
Rasavirus_16715_28	KX673223	ERR1301467	ERR1301556	Rv_2	*Rattus argentiventer*	Fecal	Unknown	14 Nov 2014	9,606	40.4	823
Rasavirus_16715_3	KX673224	ERR1301444	ERR1301533	Rv_2	*Rattus argentiventer*	Fecal	Unknown	12 Nov 2014	9,580	40.5	17
Rasavirus_16715_36	KX673225	ERR1301474	ERR1301563	Rv_1	*Rattus argentiventer*	Fecal	Unknown	14 Nov 2014	9,911	44.3	16058
Rasavirus_16715_4	KX673226	ERR1301445	ERR1301534	Rv_2	*Rattus argentiventer*	Fecal	Unknown	12 Nov 2014	9,619	40.3	14
Rasavirus_16715_43	KX673227	ERR1301481	ERR1301570	Rv_2	*Rattus argentiventer*	Fecal	Unknown	14 Nov 2014	9,562	40.4	24
Rasavirus_16715_52	KX673231	ERR1301490	ERR1301579	Rv_2	*Rattus argentiventer*	Fecal	Unknown	10 Jun 2014	8,678	40.8	27
Rasavirus_16715_52_2	KX673232	ERR1301490	ERR1301579	Rv_1	*Rattus argentiventer*	Fecal	Unknown	10 Jun 2014	8,497	44.7	50
Rasavirus_16715_57	KX673233	ERR1301495	ERR1301584	Rv_2	*Rattus argentiventer*	Fecal	Unknown	10 Jun 2014	9,599	40.3	9097
Rasavirus_16715_67	KX673236	ERR1301505	ERR1301594	Rv_2	*Rattus argentiventer*	Fecal	Unknown	11 Jun 2014	9,584	40.3	966

a Median depth of coverage was determined by mapping all quality controlled reads to the final genome using Bowtie2 with –very-sensitive-local settings. Coverage was calculated as the number of reads mapped per genome/length of genome/divided by 129.

In two human fecal samples, husaviruses (KX673274 and KX673221) showed high level of amino acid sequence identity to the previously described husaviruses KT215901, KT215902, and KT215903 (97–98% amino acid identity). In contrast, an additional husavirus detected in a human rectal swab (KX673248) showed only 25% amino acid identity over the entire polyprotein with other husaviruses.

Posavirus sequences could be detected in thirty-three (of 189) pig rectal swabs (17% frequency) and in eight (of 146) pig fecal samples (5% frequency). In each of four pig rectal swabs (sample IDs 17189_4, 17819_95, 17668_11_2, and 17668_13), two distinct strains of posaviruses were identified, while in one sample (17668_33) three distinct posaviruses were identified. The posavirus sequences identified in this study have the closest sequence identity to variants detected in farmed pigs in the USA (Shan et al. 2011, Hause et al. 2015, 2016). Moreover, novel posa-like genome sequences were found in nine (of 45) rat fecal samples [provisionally named rat stool-associated RNA viruses (rasaviruses)], and in thirteen (of 135) bat fecal samples [provisionally named bat stool-associated RNA viruses (basaviruses)]. In one rat (16715_47) and in three bat fecal samples (16715_52, 16715_61, and 16715_71) two distinct rasa/basaviruses were identified.

**Figure 1.**
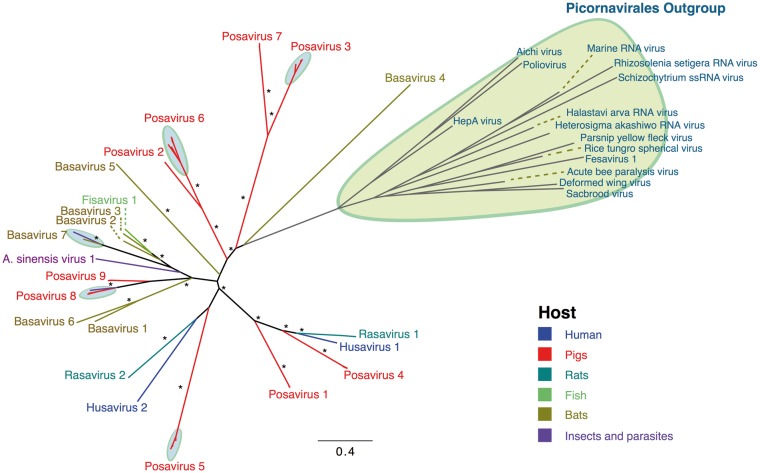
Maximum-likelihood phylogenetic tree of the predicted RdRP protein domain. The maximum-likelihood phylogenetic tree was constructed in IQtree under the LG + G+I amino acid substitution model as the best-fitted model with 500 pseudo-replicates. The tree was visualized in FigTree1.4.2. Branches were colored according to the enteric samples from the hosts in which viruses were identified (blue: human, red: pig feces, dark green: rat feces, light green: fish intestinal content, brown: bat feces, and purple: insects and parasites). Significant bootstrap values (>80) are indicated with an *.

**Figure 2.**
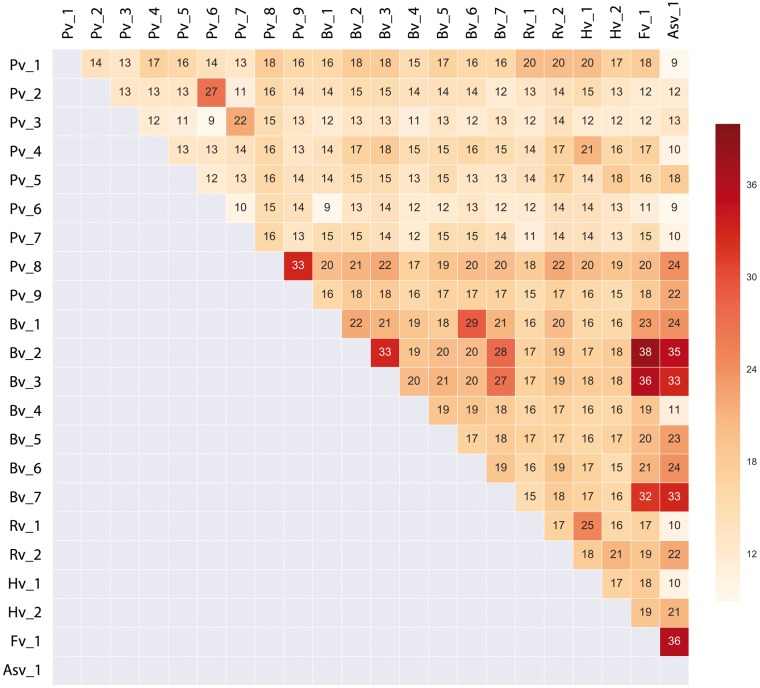
Average percentage of amino acid identity across PPLVs lineages. The amino acid sequences of the complete polyprotein of different lineages were individually aligned against each other using the ClustalW in Geneious. The numbers on the *x*- and *y*-axes represent the different lineages (for more detail see Supplementary Table S1).

The low level of shared nucleotide identity between these novel viruses made it difficult to perform phylogenetic analyses at the whole genome level. Therefore, the protein sequence encoding the most conserved region, a provisional RdRP protein, was identified and used for phylogenetic analysis. This analysis supported a conclusion that two husaviruses (KX673221 and KX673274) belonged to a lineage that includes the previously described husaviruses (KT215901, KT215902, and KT215903), while husavirus KX673248 was distant (Fig. 1). Based on this phylogenetic analysis and using a pairwise amino acid identity cutoff of 40%, twenty-two lineages could be identified. While most lineages were found in only a single source type of sample (e.g. all porcine), the Bv_7 lineage comprised basaviruses and a virus isolated from a fruit fly *Drosophila subobscura* and the two lineages Pv_8 and Pv_9 comprised posaviruses and viral sequences derived from a parasite (*Ascaris suum* ; Shan et al. 2011) (Fig. 1). For each lineage, a representative virus genome (based on the most complete sequence of the twenty-nine sequences present in the GenBank database and the newly identified sequences) was selected and characterized in more detail (Supplementary Table S1). The length of these genomic sequences ranged from 8,576 nt to 11,318 nt with a G + C content of 31.0–53.0%. The twenty-two lineages share, on average, only 9–38% amino acid identity across the entire polyprotein (Fig. 2).

Phylogenetic analysis was also performed on amino acid sequences encoding the conserved helicase, 3C protease, capsid I and capsid II domains. Due to the high sequence diversity, each set of sequences was trimmed to the most conserved region of each identified conserved domain.

Neighbor-joining (NJ) and maximum-likelihood (ML) trees were constructed individually for each of the conserved domain set of sequences. The NJ tree topology of the RdRp (left panel Supplementary Fig. S1A) was relatively consistent with the ML tree topology of RdRP (Fig. 1). However, this relatively consistency was not observed in the NJ versus ML trees in other domains (left panel compared right panel, Supplementary Fig. 1), probably due to the great sequencing divergence hence challenging proper ML tree inference.

The prevalence of husaviruses among stools samples from Vietnamese individuals was 1.4% (1/71) in healthy human rectal swabs and 0.3% (2/573) in human diarrheal feces. Rasaviruses and basaviruses were detected in 22 and 9% of the rat and bat fecal samples, respectively. Pigs also commonly carry these viruses, with posavirus being found in 17% of the rectal swabs and in 5% of the fecal samples examined in this study. The frequency of virus detection was significantly higher in rectal swabs compared to fecal samples for posaviruses (*P* value = 0.002; Chi-squared test). The frequency of husavirus positive samples was too low to draw conclusions about the prevalence in rectal swabs compared to fecal samples (*P* value = 0.59; Fishers’ Exact test).

**Figure 3.**
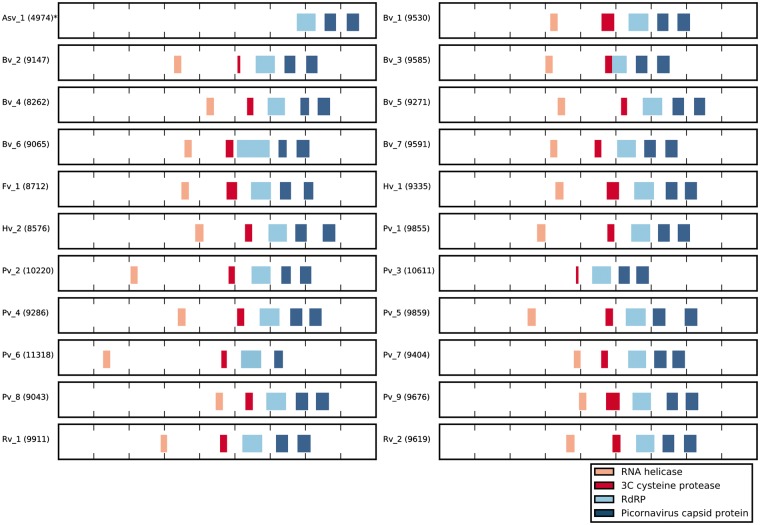
Genomic organization of different identified PPLVs lineages. The putative conserved protein domains, as determined by a conserved domain search (see ‘Methods’), and their relative position in the viral genome are illustrated. The number next to the lineage name indicates the length of each genome, the Asv1 genome (marked with an *) was only partially (<5,000 nt) sequenced. Peach blocks indicate the presence and position of an RNA helicase domain (superfamily cl21455), red blocks indicate the 3C cysteine protease domain (superfamily cl02893), light blue blocks indicate an RNA-dependent RNA polymerase (RdRP) domain (superfamily cl02808) and dark blue blocks indicate picornavirus capsid domain (superfamily cl13999). All identified conserved domains are drawn in scale related to their genome size.

While members of the *Picornavirales* typically contain a Hel-Pro-Pol replication block (Le Gall et al. 2008), some of the recently identified posaviruses initially appeared to not encode a recognizable conserved protease domain (Shan et al. 2011; Hause et al. 2015, 2016). A local HMMER search (Eddy 2011) using the complete PFAM library (Marchler-Bauer et al. 2015) failed to identify a recognizable picornavirus 3C protease domain in the majority of genomes. It was unlikely that these viruses completely lacked the protease and we suspected that the failure to detect the protease domain could be due to sequence diversity in the protease domain of RNA viruses (Koonin and Dolja 1993). Accordingly, a refined 3C protease HMM profile was constructed including all newly identified protease domains in posaviruses. A search using this refined protease domain profile identifed a putative protease domain in all of the posaviruses (Supplementary Table S1). In addition, all genomes were found to encode an RNA helicase domain, an RdRP domain and two picornavirus capsid domains [with the exception of Asv_1 since this GenBank entry is only partial and posavirus_3 where no conserved RNA helicase domain could be identified (Fig. 3)].

**Figure 4.**
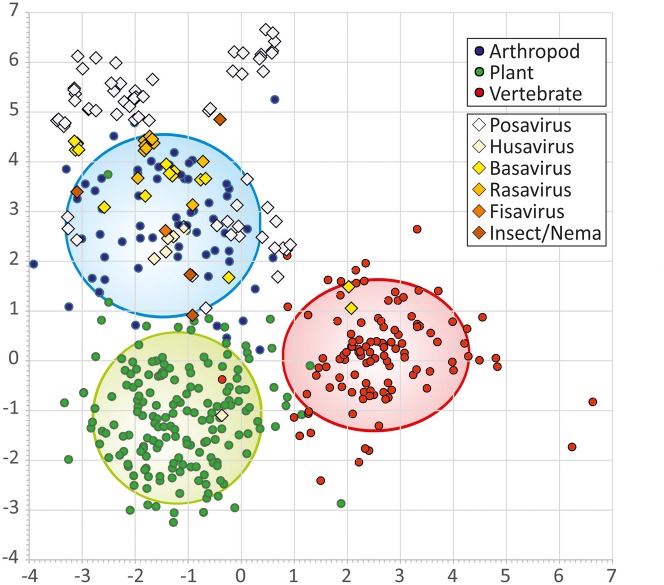
Discriminant analysis of the dinucleotide bias in PPLVs compared to members of the *Picornaviridae* for which the infectious host is known. Viruses infecting arthropods are indicated with blue circles, viruses infecting plants in green circles and viruses infecting vertebrates in red circles. The lines indicate the 95% interval. Posaviruses are plotted in white diagonals, husavirus in light yellow, basavirus in yellow, rasavirus in dark yellow, fisavirus in orange and insect/nematode infecting viruses in dark orange.

The G + C contents for all PPLV sequences were determined but no specific G + C content pattern was observed in virus sequences from different hosts. The husaviruses showed the highest G + C content (50.5–53.0%), followed by posaviruses (30.9–51.2%), rasaviruses (40.5–44.0%), and basaviruses (32.2–48.2%) (Table 1 and Supplementary Table S1). As previously described (Kapoor et al. 2010), nucleotide composition analysis (NCA) can be used to predict the host range of members of Supergroup 1 RNA virus, that includes the *Picornavirales*. Sequences from 105 PPLV genomes obtained in the current study and from published sources were analyzed using a pre-trained dataset of reference genomes from three categories of hosts (arthropod, plant, and vertebrate; Fig. 4). The analysis revealed that almost all posaviruses as well as all husa-, basa-, rasa-, fisa-, insect-, and nematoda viruses clustered within the arthropod group (Fig. 4). Two basaviruses (lineage Bv_2) cluster within the vertebrate group of the *Picornaviridae* and one husavirus (lineage Hv_2) clustered within the plant virus group of the *Picornaviridae*. These observations fell within the 5% error range of the analysis (95% prediction accuracy of the controls, Supplementary Table S2).

## 3. Discussion

Here we report the identification of new *Picornavirales* members related to sequences previously identified in pig stool (posavirus) and human stools and/or rectal swabs (husavirus). In addition, we describe newly identified bat stool-associated viruses (basaviruses) and rat stool-associated viruses (rasaviruses) which have a similar genomic organization compared to posaviruses. Posaviruses are known to be widely distributed geographically with examples found in the USA (Shan et al. 2011) and in China (Zhang et al. 2014), however, this is the first detection of husavirus in human stools outside the Netherlands. These Posa and Posa-like virus genomes are collectively referred as PPLVs.

The PPLV genomes were identified based on identity to previously identified posaviruses and their lack of close protein homology to any of the *Picornavirales* families and the presence of a set of five protein functional domains. Using standard phylogenetic analyses, the PPLVs formed lineages which are distinct from the five established *Picornavirales* families (*Dicistroviridae*, *Iflaviridae*, *Marnaviridae*, *Picornaviridae* and *Secoviridae*) and the unassigned *Picornavirales* genome sequences. However, there is as much diversity between the PPLVs as there is between the PPLVs and the established *Picornavirales* families.

We used the USEARCH clustering algorithm in an attempt to determine how close the PPLV genomes are to existing *Picornavirales* genomes. All 108 PPLV genome sequences were combined with all available *Picornavirales* full genome sequences from GenBank (5,766 genomes, excluding those with stretches if Ns greater than 20). At various levels of homology (ranging from 60% to 90% nucleotide identities), the PPLV sequence clusters were distinct from the clusters formed from the *Picornavirales* genomes, i.e. there were no clusters containing both PPLV sequences and genomes classified in one of the *Picornavirales* families (results not shown). Thus we think it is valid to conclude that none of the PPLVs belong to established *Picornavirales* families. The PPLV group is however too diverse to be classified as a single virus family. Given the pace at which new virus sequences are becoming available, we believe the best approach is to deposit these sequences with a tentative identification as PPLV and as more detailed sequence data become available a better organization of these virus sequences into well-supported family or families can be made.

Members of PPLV group have now been identified in pigs, humans, fish, rats, bats, insects (*Anopheles sinensis* and *Drosophila subobscura*), and parasites (*Ascaris suum*). Based on phylogenetic analysis of the RdRP domain and pairwise comparisons of the entire polyprotein, we propose that the PPLVs comprise twenty-two phylogenetic lineages. These PPLVs could also be grouped in twenty-two lineages based on the NJ trees constructed from amino acid sequences encoding other conserved domains (putative helicase, protease, capsid I, and II, Supplementary Figs S1A–D).

Consistent with other members of the *Picornavirales*, most of the newly described PPLV sequences encoded a Hel-Pro-Pol replication block. However, in some of the genome sequences, no recognizable protease domain could be identified using conventional methods with an existing pre-made PFAM domain based on a limited number of picornavirus protease domains. However, a more detailed protease domain database based on a broader set of *Picornavirales* proteases, including the novel putative posavirus protease domains, revealed the presence of a protease domain across the entire range of PPLV genomes (Fig. 3).

In an attempt to infer a putative cellular host for the PPLVs, a nucleotide composition analysis **(**NCA) was performed. NCA incorporates composition measures of dinucleotide frequencies and has been used to predict the infectious hosts of members of RNA virus supergroup I (Kapoor et al. 2010). In a set of sequences for which the infectious host was known, the analysis was able to accurately classify viruses as either being of vertebrate, plant, or arthropod origin in around 95% of the cases (Koonin et al. 2008). Using this analysis method, the PPLV genome sequences were found to cluster with viruses from the arthropod group (Fig. 4). The two outliers of the discriminant analysis (lineage HV_2 and Bv_2) fall within the 95% confidence interval, but given their substantial sequence divergence from other PPLVs it is possible that these viruses infect another hosts.

The prevalence of husaviruses in fecal samples (0.3%) and rectal swabs (1.4%) in Vietnam was lower than the 3.5% prevalence observed in a cohort of predominantly healthy HIV-1 positive and negative individuals (Oude Munnink et al. 2015). Prevalence differences may be due to differences in RT-PCR detection versus genome assembly from next generation sequencing, the small sample numbers and/or true differences between the cohorts. Of interest, posavirus could be detected significantly more often in pig rectal swabs compared to pig fecal samples (*P* = 0.002), suggesting that the viruses are enriched on the rectal epidermis. This enrichment and the clustering of posaviruses with the arthropod viruses may be consistent. It is known that intestinal parasites can be found perirectally and can be detected using the scotch tape test (Enterobius_Vermicularis_Diagnostic_Test). An interesting follow-up analysis would be to determine the scotch tape virome and our prediction would be that members of the PPLV group can be found in these samples.

In summary, this study provides a large set of seventy-six new PPLV genomes, quadrupling the available genomic data for this broad group viruses. A novel Vietnamese husavirus genetically distant from the previously described husaviruses was identified and PPLV members were also detected in rat and bat feces. In addition, we were able to clarify two additional features of posavirus virology: a putative protease domain was detected in all PPLV genomes and NCA revealed that members of the PPLV group share a conserved nucleotide composition with viruses infecting members of the arthropod phylum.

## 4. Methods

### 4.1 Samples

Fecal material was collected from 135 bats (*Scotophilus kuhlii*), 573 humans (*Homo sapiens*), 146 pigs (*Sus domesticus*), and 45 rats (*Rattus argentiventer*). In addition, rectal swabs were collected from seventy-one humans and 189 pigs. These samples were collected from a 150 square kilometer area of Dong Thap province, a southern region within the Mekong Delta River in Vietnam. All fecal samples from human enrollees were diarrheal patients admitted to Dong Thap Provincial hospital, while human rectal swabs were taken from healthy farmers and family members. Pig fecal samples and rectal swabs were collected from individual pigs from breeding farms. Rat fecal samples were collected from rats, which were purchased on the market or collected from rice-field traps. The disease state of these animals is unknown. Bat fecal samples were collected from beneath roosting sites.

Ethical approval for the study was obtained from the Oxford Tropical Research Ethics Committee (OxTREC Approval No. 15-12) (Oxford, United Kingdom), the institutional ethical review board of Dong Thap Provincial Hospital (DTPH) and the Sub-Department of Animal Health Dong Thap province (Dong Thap, Vietnam).

### 4.2 Illumina sequencing

Fecal samples (*n* = 899) or rectal swabs (*n* = 260) were centrifuged for 10 min at 10,000 × g after which the samples were DNase treated at 37 °C for 30 min (20 U of TURBO DNase, Thermo Fisher per 100 µl of sample). Nucleic acids were extracted, transcribed into cDNA and subjected to second strand synthesis (de Vries et al. 2011, 2012). The resulting dsDNA from each sample was sheared and fractionated to 400–500 bp in length after which Illumina adapters with a unique barcode were ligated to the fragments. Resulting libraries were sequenced with the Illumina MiSeq or HiSeq platforms to generate 1–2 million 150 nt (MiSeq) or 3–4 million 250 nt (HiSeq) paired-end reads per sample.

### 4.3 *De novo* assembly and complete genome characterization

Adaptor sequences were removed and sequence reads that passed quality control were *de novo* assembled using SPAdes version 3.5.0 (Bankevich et al. 2012) followed by improve_assembly (Page 2012). The resulting contigs were subjected to a modified protein blast search using USEARCH (Edgar 2010) to identify novel members of the *Picornavirales*. To minimize the effects of Illumina cross-talk, all preliminary contigs were examined and contigs within a sample with low median coverage (greater than 10-fold lower than the major contig in the sample) were excluded from the analysis. For all PPLVs reported here, the complete or nearly complete (>8,000 nt) genome was obtained and for all viruses the genome coverage was determined by mapping all quality controlled sequence reads to the final genome. The G + C content was determined using Geneious (Kearse et al. 2012). To determine the average percentage amino acid identity across the PLLV lineages, amino acid sequences were aligned using the ClustalW in Geneious (Kearse et al. 2012).

To identify conserved protein domains encoded by the new genomes an RPS-BLAST search (Marchler-Bauer et al. 2015) against the Conserved Domain Database (CDD) was performed. The initial screen identified the helicase, RdRP, and picornavirus capsid (I and II) domains across almost all genomes. However, the 3C protease domain was identifed in only a subset of genomes, suggesting either a true absence or a mis-identification due to great sequence divergence. A modified 3C protease domain profile was generated from a protein sequence alignment of the conserved domain (pfam00548) from the CDD and used to identify the 3C protease-like regions in the new PPLV genomes. An updated alignment containing all the putative protease domains used to create a new HMM index file. A local hmmsearch analysis with this updated 3C protease profile was then performed to identify divergent putative protease domains in the PPLV genome sequences.

### 4.4 Discriminant analysis of the dinucleotide bias

Nucleotide composition analysis (NCA) was performed as previously described (Kapoor et al. 2010) using sequences of members of RNA virus supergroup 1 (Koonin et al. 2008) infecting vertebrates (*n* = 113), arthropods (*n* = 66), and plants (*n* = 172) for classification. The frequencies of each mononucleotide and dinucleotide were used for discriminant analysis to maximize discrimination between control sequences; these canonical factors were then used to infer the host origin of the RNA virus sequences obtained in the current study.

### 4.5 Phylogenetic analysis

All PPLV sequences identified in this study combined with all complete PPLV genomes present in the GenBank database (retrieved on 16 July 2016) were aligned using muscle (Edgar 2004). Amino acids sequences were trimmed to the region encoding for the conserved domains and alignments were manually inspected and trimmed to the most conserved part. Phylogenetic analyses were performed on the conserved putative conserved domains using IQtree (Nguyen et al. 2015), under the best-fitted amino acid model with 500 pseudo-replicates. The resulting trees were visualized using FigTree v1.4.2 (http://tree.bio.ed.ac.uk/software/figtree/).

### 4.6 Statistical analysis

Statistical analysis was performed using the two by two table from Open Epi (Sullivan et al. 2009). As a measure of association, the Chi-squared test or the Fishers’s exact test was used.

### 4.7 GenBank accession numbers

All PPLV genome sequences generated in this study were deposited into the GenBank database under the accession numbers KX673215–KX673290.

## Supplementary Material

Supplementary Figure 1

Supplementary Table 1

Supplementary Table 2
