# Supplementary figures and images for: Characterization of Posa and Posa-like virus genomes in fecal samples from humans, pigs, rats, and bats collected from a single location in Vietnam
Source: Virus Evol. 2017 Aug 23;3(2):vex022. doi: 10.1093/ve/vex022 (PMC5597861; doi:10.1093/ve/vex022)

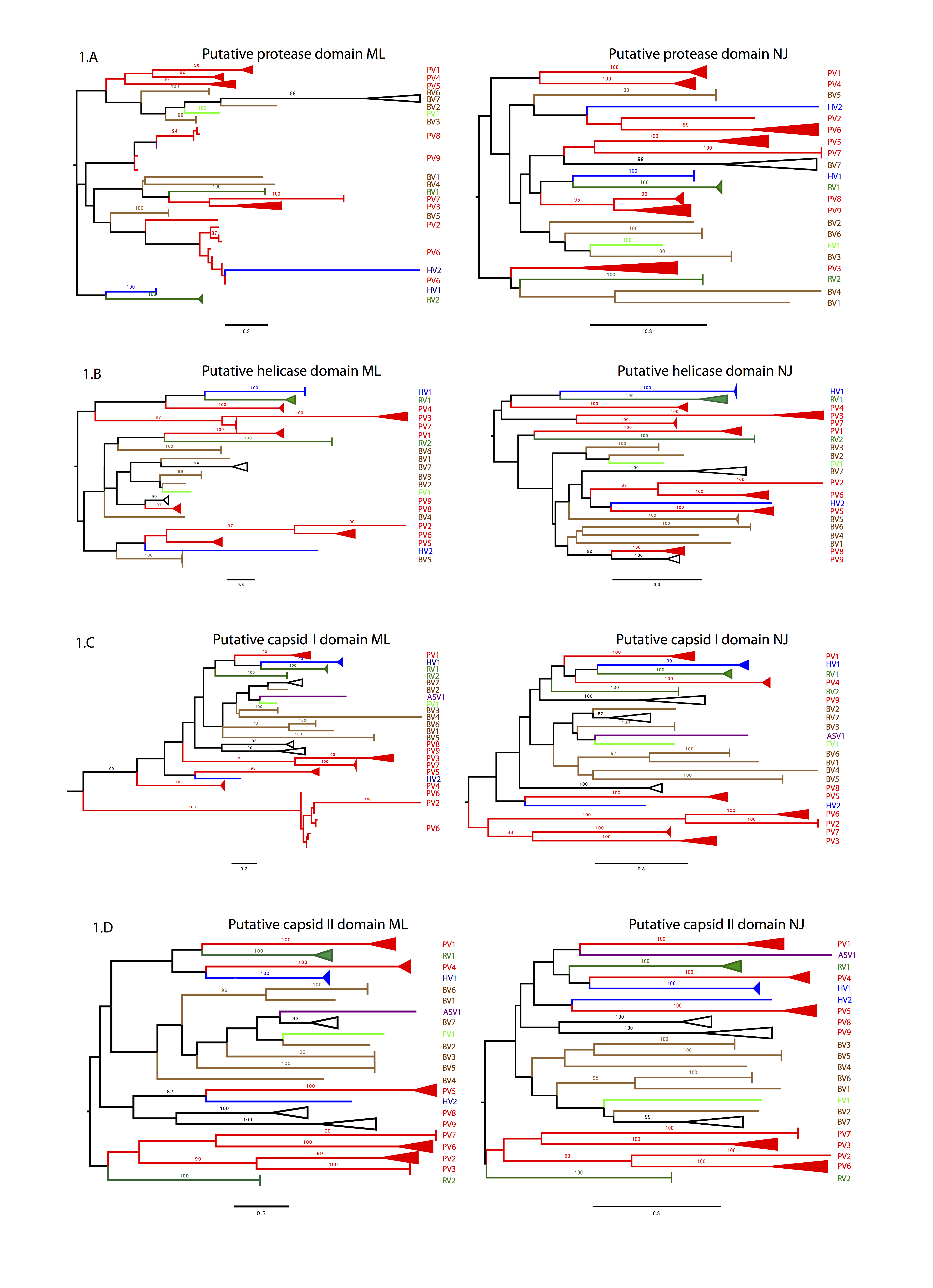

Supplement: Supplementary Figure 1 [file vex022_supp_figure_1.jpeg]
